# Single Sensillum Recordings in the Insects *Drosophila melanogaster* and *Anopheles gambiae*

**DOI:** 10.3791/1725

**Published:** 2010-02-17

**Authors:** Maurizio Pellegrino, Takao Nakagawa, Leslie B. Vosshall

**Affiliations:** Laboratory of Neurogenetics and Behavior, The Rockefeller University

## Abstract

The sense of smell is essential for insects to find foods, mates, predators, and oviposition sites^3^. Insect olfactory sensory neurons (OSNs) are enclosed in sensory hairs called sensilla, which cover the surface of olfactory organs. The surface of each sensillum is covered with tiny pores, through which odorants pass and dissolve in a fluid called sensillum lymph, which bathes the sensory dendrites of the OSNs housed in a given sensillum. The OSN dendrites express odorant receptor (OR) proteins, which in insects function as odor-gated ion channels^4, 5^. The interaction of odorants with ORs either increases or decreases the basal firing rate of the OSN. This neuronal activity in the form of action potentials embodies the first representation of the quality, intensity, and temporal characteristics of the odorant^6, 7^.

Given the easy access to these sensory hairs, it is possible to perform extracellular recordings from single OSNs by introducing a recording electrode into the sensillum lymph, while the reference electrode is placed in the lymph of the eye or body of the insect. In *Drosophila*, sensilla house between one and four OSNs, but each OSN typically displays a characteristic spike amplitude. Spike sorting techniques make it possible to assign spiking responses to individual OSNs. This single sensillum recording (SSR) technique monitors the difference in potential between the sensillum lymph and the reference electrode as electrical spikes that are generated by the receptor activity on OSNs^1, 2, 8^. Changes in the number of spikes in response to the odorant represent the cellular basis of odor coding in insects. Here, we describe the preparation method currently used in our lab to perform SSR on *Drosophila melanogaster* and *Anopheles gambiae*, and show representative traces induced by the odorants in a sensillum-specific manner.

**Figure:**
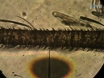


## Protocol

### 1. Odor dilutions

Most odorants are soluble in paraffin oil. However, DMSO or ethanol can also be used as alternative solvents for particular odors. Prepare appropriate dilutions (e.g. 1:10 volume : volume; v:v) from pure odorants in glass vials. Most odor dilutions are stable at room temperature, but for highly volatile compounds it is better to make working dilutions on a weekly basis. Each sensillum responds to different odors within a different concentration range. For *Drosophila*, a useful look-up table for appropriate concentrations to use with a given sensillum can be found in ^6, 7^. For the video experiment, we use paraffin oil as a solvent control and methyl acetate (10^-6^ v:v in paraffin oil) and 1-octen-3-ol (10^-7^ v:v in paraffin oil) for *Drosophila* and *Anopheles* recordings, respectively.
Using scissors, cut the chromatography paper into 3 mm x 5 cm strips so that they will fit inside Pasteur pipettes.
Pipette 30 μL of the desired odor on a filter paper strip and insert it into the glass pipette. Cut ~3 cm of air line tubing and insert it into the open end of the pipette, closing it with a connector. The connector is used to seal the pipette that will be then attached to the air-line tubing of an air pump when it is time to deliver the odor during the experiment.

### 2. Odor delivery system

Using a small drill, cut a 10 mL plastic serological pipette (e.g. at the 4 mL mark) and create two holes (e.g. at the -1.5 mL and -0.5 mL marks) that will be used to hold the pipettes containing the odors. Insert a 200 μL pipette tip in the barbed coupler and introduce the coupler into the blunt end of the 10 mL pipette. The pipette will be used as part of the delivery system for odorants.
Attach the pipette on a magnetic stand with a pipette clamp and position it near the microscope.

### 3. Sharpening electrodes

To sharpen electrodes, prepare a 0.5 M solution of potassium hydroxide (KOH) and filter it to remove fine particles (e.g. using a 45 μm filter). Take a 20 mL syringe and make a small hole (~2 mm diameter) using a needle on the wall close to the tip (~1 cm from the tip), into which the electric wire is inserted (Figure 1A).
Fill the syringe with 0.5 M KOH, and clamp it on a stand under the microscope so that the tip is placed in the field of view (Figure 1B, C). Insert the electric wire into the small hole on the syringe wall (Figure 1B), making sure that the wire is not directly in front of the entrance of the syringe, and connect this wire to the anode of the power supply.  Attach the electrode holder shaft onto the manual micromanipulator on the right side of the microscope, and attach an electric cable at the base of the electrode holder shaft with a crocodile clip, and connect the cable to the cathode of the power supply (Figure 1A). This creates an electrical circuit between the electrode holder and the syringe once the tungsten wire is inserted into the syringe (Figure 1A).
Insert the tungsten wire (~5 cm length) into the electrode holder, and attach it to the electrode holder shaft on the manipulator. Set the power supply to 6 V, and insert the tip of the wire into the syringe repeatedly to hone it, being careful to monitor the tip under the microscope during the process (Figure 1C). To get an ideal electrode for recording, place 90% of its length in the solution for up to 1 min, and pull it out slowly. Then insert only ~50% of the electrode to further thin for 30 s, and repeat it to get the tip of the wire sharpened (~10 times). The tip of the electrode should be fine enough to enter into the sensillum, but not so fine as to bend when it touches it during the recordings (steps 6 and 7). Although watching the electrode tip under the dissecting microscope while it is being sharpened is a good indication of its thickness, only looking at it under the recording microscope at high magnification will give a clear idea whether a given electrode is suitable for recording.

### 4. Insect prep: *Drosophila* antenna

Build a fly aspirator. Cut a piece of air line tubing long enough to hang comfortably around your neck (about 90 - 120 cm). Cut the tip of a 200 μL pipette tip and insert it into one end of the tube. On the other end, position a ~1.5 cm x 1.5 cm piece of mesh so that it creates a physical barrier but does not prevent air from flowing out of the tube. Cut the tip of a 1 mL pipette tip and position the wider end on top of the tube opening, blocking the mesh in between. This portion will be used to pick up and manipulate adult vinegar flies (Figure 2).
Take a microscope slide and place a piece of dental wax roughly in the middle of the long side. On top of it position a cover glass slightly tilted upwards (~30 ), making sure that the wax is not directly underneath the uppermost part, which would prevent visualization of the vinegar fly under the microscope. Pull a glass electrode with the vertical puller. Its tip should be thin and flexible enough to fit in between the second and third antennal segment, and keep the antenna stable for the recordings. Position the glass electrode on another piece of wax and place it on the side of the cover glass, far enough that when the tip is lowered it reaches the corner of the cover glass (Figure 3A).
Working from a bottle or vial of adult flies of the desired genotype, pick up an adult vinegar fly using the fly aspirator. Although females are usually used because of their bigger size, males can also be used. Place a 200 μL pipette tip on top of the 1 mL tip to avoid the fly from escaping. Blow into the tube, so that the fly is pushed towards the end of the 200 μL pipette tip. Trim the wide end of the pipette tip with the razor blade a few millimeters away from the fly itself, then insert some wax to prevent the fly from backing out. Under a microscope, cut again near the head of the vinegar fly, paying attention not to damage the animal. With a small pipette tip, push the wax to force the fly head out so that about half of the eyes extrude from the tip (Figure 3B). Make sure that its legs do not come out as well, or they might move and interfere with the recordings.
Mount the fly on a piece of wax, its head facing upwards, and place it on the slide in front of the cover glass. Push the head slightly against the corner of the glass, so that the antennae extend and rest on the glass. Lower the tip of the glass capillary in between the second and third antennal segment (Figure 3B).
Different parts of the antenna will give access to different sensilla types. Depending on particular experimental needs, the antenna will need to be oriented differently to allow access to different sensilla types. To record from large basiconic sensilla as in our example, the arista is pushed down on the glass (Figure 3B).

### 5. Insect prep: *Anopheles* maxillary palps

Using an electric aspirator (Figure 2E), collect 40 to 60 3-5 days old mosquitoes (mixed males and females) in the small plastic cage (Figure 2F). Mosquitoes should have been reared under normal conditions, i.e. in an insect incubator or insectary at 25-28°C with 70-80% humidity. Place the small plastic cage with the animals on ice for ~15 min until they are anesthetized by cold. Once the animals have stopped moving, transfer only 4-6 animals to the stage under the microscope at any one time, keeping the rest of the animals on ice during the procedures. Although females are usually used because of their sensitivity to CO_2_, males can also be employed. Select female or male mosquitoes, judging by the structure of their antenna (feather-like in females, filament-like in males), and remove their wings and legs with fine forceps to immobilize them. Keep them in a small plastic cup with a wet paper at the bottom, to prevent them from desiccating.
Put two pieces of double-sided tape (~1 cm in length) parallel to each other (~1 cm apart), at the center and at the side of the slide glass (Figure 3C). Using fine forceps, place one mosquito on the central tape under the dissecting microscope, and turn it sideways and stick its body and one eye on the tape.  Adjust the position of the maxillary palps so that both palps extend parallel on the tape (Figure 3D). Fix the maxillary palps by placing thin strings (e.g. human hairs) both at the base and at the tip of palps (Figure 3D). The side tape is used as a repository for thin strings, while the central tape needs to be replaced after every recording (Figure 3C).

### 6. Recording   *Drosophila melanogaster*

Place the slide under the microscope (Figure 4A) at low magnification and position the antenna roughly in the middle of the field of view (FOV) (Figure 4B). Gently lower the electrodes so that the reference electrode is located near the eye of the fly and the recording electrode is near the antenna (Figure 4C). Increase the magnification and re-position the antenna in the middle of the FOV (Figure 4D).
Place the odor delivery device close to the head of the fly, pointing at the antenna.
At low magnification (Figure 4C), insert the reference electrode into the eye of the fly. Lower the recording electrode on top of the antenna without touching its surface.
Switching to high magnification, control the recording electrode with the micromanipulator and insert it into a selected sensillum (Figure 4D). Any point along the sensillum length is good for recording. Once inside the sensillum, the electrode can be pushed farther in (sometimes all the way through) to obtain a better signal to noise ratio. Once the electrode is in the sensillum, the spontaneous activity of the cells can be detected.

### 7. Recording   *Anopheles gambiae*

Place the glass slide under the microscope at low magnification (10x) and position the maxillary palps roughly in the middle of the FOV and the head at the top (Figure 4E). Rotate the stage until one of the palps is at a right angle with the recording electrode.
Adjust the height of the electrodes so that the reference electrode is positioned just above the eye of the mosquito and the recording electrode is near the maxillary palps.
Place the odor delivery device so that it is as close as possible to the palps.
Insert the reference electrode into the eye at lower magnification (10x), and switching to the higher magnification (100x), insert the recording electrode into the peg sensillum on the palp (Figure 4F). Once the electrode is in the sensillum, the spontaneous activity of the cells can be detected.

### 8. Representative results

Depending on the sensillum and the quality of the recording, one can distinguish different numbers of olfactory neurons within a single trace. In the large basiconic sensilla of *Drosophila melanogaster*, for example, between 2 and 4 cells that differ in spike amplitude should appear during the recording ^9, 10^.

In our video experiment, the *Drosophila* ab2 sensillum shows two cells, an A cell (Figure 5, blue spikes) and a B cell (Figure 5, green spikes). Neither cell is activated during application of paraffin oil (Figure 5A), while only the A cell responds to the 10^-6^ dilution of methyl acetate (Figure 5B).

In the maxillary palp of *Anopheles gambiae*, the grooved peg sensillum contains three cells, but only two are easily discriminated (Figure 5C, blue and green spikes, respectively). In the video experiment we show how the B cell responds to a 10^-7^ dilution of 1-octen-3-ol (Figure 5D).


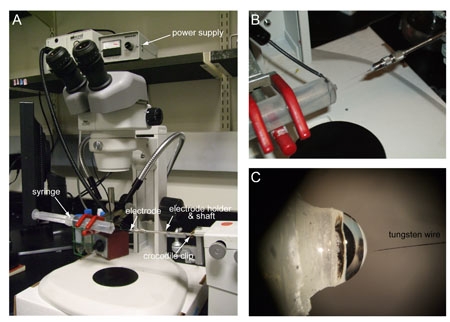
 **Figure 1. Electrode sharpener** **(A) **General view of the electrode sharpener apparatus. **(B)** The syringe containing 0.5 M KOH (left) used to sharpen the electrode (right). **(C)** Close-up of the electrode tip next to the opening of the syringe.


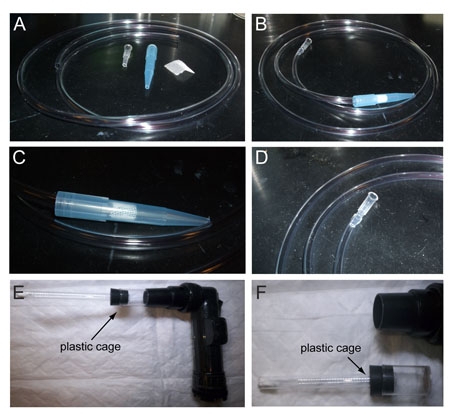
 **Figure 2. How to prepare a fly aspirator and mosquito aspirator** **(A)** Starting material: air line plastic tubing, two cut pipette tips, and mesh. **(B)** The fly aspirator once it is completed. **(C)** Detail of the end that is used to catch vinegar flies. **(D)** Detail of the other end of the fly aspirator. **(E)** The electric aspirator for mosquito collection consists of a main body and a detachable plastic cage. **(F)** The detachable plastic cage for mosquitoes.


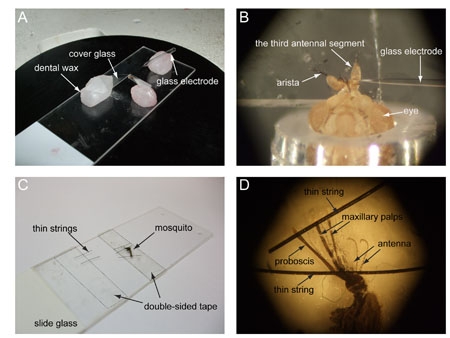
 **Figure 3. Preparing a vinegar fly (*Drosophila melanogaster*) and a malaria mosquito (*Anopheles gambiae*) for recording** **(A)** Picture of a vinegar fly mounted on the slide before positioning it under the microscope. **(B)** Close-up of the vinegar fly head with the antenna kept in place by the glass capillary. **(C)** Picture of a mosquito mounted on the slide. **(D)** Close-up of the mosquito head with proboscis and palps sticking on the tape.


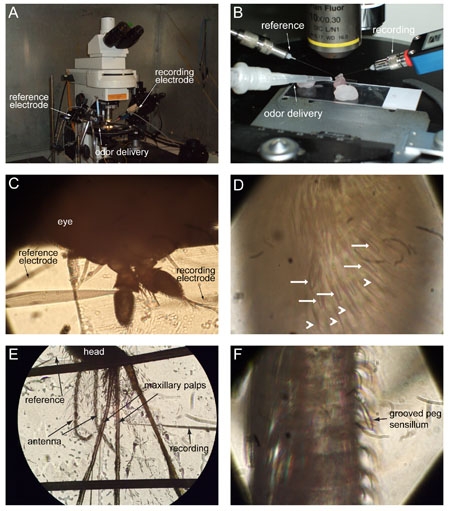
 **Figure 4. Recording from a vinegar fly (*Drosophila melanogaster*) and a malaria mosquito (*Anopheles gambiae*)** **(A)** View of the electrophysiology setup. **(B)** Close-up of the fly preparation mounted on the microscope. Notice the respective position of the recording electrode (left), the odor delivery system (middle pipette), and the recording electrode (right). **(C)** Image of the fly under the 10x objective. **(D)** Image of the fly antenna under the 100x objective; big basiconic sensilla (arrows), interspersed among non-sensory hairs (arrowheads). **(E)** 10x view of a mosquito mounted for recording. **(F)** High magnification view of the mosquito palp and a peg sensillum (arrow).


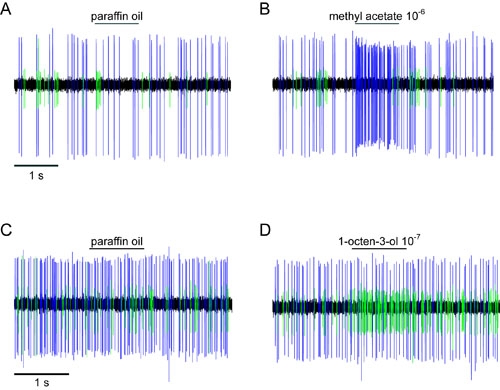
 **Figure 5. Examples of recordings from *Drosophila melanogaster* and *Anopheles gambiae*** **(A)** The ab2 sensillum of *Drosophila melanogaster* houses two sensory neurons; the A cell (blue spikes) and B cell (green spikes). **(B)** The A and B cells during application of 10^-6^ methyl acetate. **(C)** The peg sensillum of *Anopheles gambiae* houses two sensory neurons; the A cell (blue spikes) and B cell (green spikes). **(D)** Application of 10^-7^ 1-octen-3-ol to the peg sensillum.

## Discussion

Olfactory cues are used by organisms to identify food sources, potential mates, and predators. Olfactory sensory neurons (OSNs) are the first relay center between external stimuli and higher centers of the brain where the information is further processed. In *Drosophila melanogaster* and *Anopheles gambiae*, OSNs are easily accessible and their electrical activity can be monitored while stimulated by odor puffs.

The single sensillum recording (SSR) technique explained in this video has been widely used to record from OSNs and study their electrical responses to a large number of odorants^6, 7^. The deorphanization of olfactory receptors (ORs)^6, 11^ and the mapping of ORs to specific locations on the *Drosophila* antenna^9, 12, 13^ has made the SSR technique a powerful tool to analyze the electrophysiological properties of specific ORs *in vivo*, as a first step to understand how the external olfactory world is translated into electrical signals through its OSNs and eventually perceived by the animal.

## References

[B0] Boeckh J (1962). Elektrophysiologische Untersuchungen an einzelnen Geruchsrezeptoren auf der Antenne des TotengrAbers (Necrophorus Coleoptera). Z. Vergl. Physiol.

[B1] Schneider D, Hecker E (1956). Zur Elektrophysiologic der Antenne des Seidenspinners Bombyx mori bei Reizung mit angereicherten Extrakten des Sexuallockstoffes. Z. Naturforschg.

[B2] Touhara K, Vosshall LB (2009). Sensing odorants and pheromones with chemosensory receptors. Annu Rev Physiol.

[B3] Sato K (2008). Insect olfactory receptors are heteromeric ligand-gated ion channels. Nature.

[B4] Wicher D (2008). Drosophila odorant receptors are both ligand-gated and cyclic-nucleotide-activated cation channels. Nature.

[B5] Hallem EA, Ho MG, Carlson JR (2004). The molecular basis of odor coding in the Drosophila antenna. Cell.

[B6] Hallem EA, Carlson JR (2006). Coding of odors by a receptor repertoire. Cell.

[B7] Boeckh J, Kaissling KE, Schneider D (1965). Insect olfactory receptors. Cold Spring Harb Symp Quant Biol.

[B8] de Bruyne M, Foster K, Carlson JR (2001). Odor coding in the Drosophila antenna. Neuron.

[B9] Lu T (2007). Odor coding in the maxillary palp of the malaria vector mosquito Anopheles gambiae. Curr Biol.

[B10] Hallem EA, Fox AN, Zwiebel LJ, Carlson JR (2004). Olfaction: mosquito receptor for human-sweat odorant. Nature.

[B11] Couto A, Alenius M, Dickson BJMolecular (2005). anatomical, and functional organization of the Drosophila olfactory system. Curr. Biol.

[B12] Fishilevich E, Vosshall LB (2005). Genetic and functional subdivision of the Drosophila antennal lobe. Curr Biol.

